# Prostatic Diseases, with Special Reference to the Prostate of Old Age1Read before the Buffalo Academy of Medicine, December 5, 1893.

**Published:** 1894-02

**Authors:** Marcell Hartwig

**Affiliations:** Buffalo, N. Y.


### PROSTATIC DISEASES WITH SPECIAL REFERENCE TO THE PROSTATE OF OLD AGE.[1]

By MARCELL HARTWIG, M. D., Buffalo, N. Y.

The deficiency of my knowledge in regard to the subject chosen for an introduction to the discussion of tonight has made me grasp it with eagerness, in the hope of eliciting for myself something worth the while in practice, by searching the literature ; but, I am sorry to say that my somewhat sanguine hopes have been sadly disappointed. In spite of all endeavors and surgical daring, especially fostered and developed in the last few years, incidental to the spread of antisepsis and asepsis, with their marvelous advances and results, the benefits from operating in prostatic hypertrophy have remained very limited indeed. I am jumping in *medias res.* Let us first duly consider the rest of prostatic diseases. We may divide them in: (1) Faults of prime formation ; (2) cuts, bruises and hemorrhage ; (3) acute purulent prostatitis ; (4) chronic prostatitis and prostatorrhea ; (5) tuberculosis ; (6) atrophy ; (7) tumors, especially cancers ; (8) stones in the prostata; (9) neurosis (respectively fissures of the prostate) (Home); (10) hypertrophy and fibromyomata.

I. Faults of prime formation of surgical importance are cysts, probably retentive cysts, from dilated ducts. I could not find a case which was diagnosticated before operation, although such diagnosis would be all important, because a very simple puncture should suffice. Everybody ought to think of a cyst when prostatic obstruction occurs in early age, and he may, by deftly feeling, possibly discover it by bulbous bougie and rectal palpation combined.

II. The prostate is very seldom the sole organ wounded, because of its secluded position, and if it is, the other organs which have been cut have predominant importance, still it may happen that the prostate is stabbed, and bleeds profusely. Pressure by a rectal colpeurynter is mostly sufficient. It is not to be forgotten that the blood may escape unseen into the bladder and cause profound anemia before discovered, even where we operate ourselves. So we have to remain on guard. Israel has reported a most astounding case, a few years ago. Where doubt exists, the bladder should be catheterized, a small stab wound in the perineum enlarged, and, if danger is apparent, suprapubic incision added, and the prostate cauterized, or tamponed from above.

1. Read before the Buffalo Academy of Medicine, December 5, 1893.

Trendelenburg's posture is the most advantageous for seeing from above the pubis.

III. The causes of acute purulent prostatitis are somewhat dark yet. This is a field where bacteriological research will earn some laurels yet in coming times. Gonorrhea seems a frequent cause. Wound infection is another, but some cases have hitherto defied explanation (my case). The course of the disease is varied. In the majority of cases, the abscess ruptures into the urethra, but sometimes into the perineum, exceptionally above the fascia profunda, and then it may extend behind the peritoneum till to the diaphragm, and is most likely to end in death. Fever is sometimes high, chills frequent, pain, of course, located in the perineum. Inability to urinate appears early. Diagnosis ought to be made as early as possible, and incision from within the urethra ditto. Only when rectal or perineal edema exists already, I doubt whether intraurethral incision will suffice, as there may exist already paraprostatic abscess, which, by all means, demands perineal incision. As a matter of fact, the majority of abscesses broke spontaneously into the urethra, those which the surgeons incised were all attacked from the perineum. The proceeding needs no description; it could be questioned only whether to incise in the rhaphé or by a cut around the front of the rectal sphincter. The latter incision gives more space, and I should prefer it, if suspecting that the fascia profunda is broken and the retroperitoneal space infected.

IV. Chronic prostatitis may be purulent and form an abscess which can be detected by rectal and combined palpation, and, of course, incised, as above described, for acute cases, but, as a rule, this form is characterized merely by a feeling of heaviness in the perineum, some pain in ejaculation, and prostatorrhea. Patients with chronic prostatitis are inclined to hypochondria and neurasthenia. The cause is almost always gonorrhea or self-abuse. The differential diagnosis is by no means easy, and many cases remain forever dubious. The size and feel of the prostate show no distinctive feature; the only means of diagnosis lie in the microscopical examination. Azoospermia and urethritis posterior are most likely mistaken for chronic prostatitis. The posterior urethritis carries more cells resembling pus. Azoospermia is *mostly* connected with want of erections. Spermatorrhea carries the characteristic sperma. Catarrhal condition of Littre's and Cowper's glands shows a glassy appearance of the secretion, while the prostatic juice is milky, has the characteristic odor (not the sperma), and contains globules, which some call amyloid, while Posner thinks to have it proven that they are lecithin. The most unmistakable proof can be had by producing the Boettcher-Charcot crystals. One drop of the secretion is mixed with one drop of a one per cent. solution of phosphate of ammonia, and after standing under the coverglass for a while, shows the crystals, the base of which is $C^2 H^5 N^6$. The simplest way of obtaining the prostatic juice is by expressing the prostate from the rectum after urinating. I have found that the quantity in a case of chronic prostatorrhea was largely diminished the day after a night-emission. Treatment must be tonic. The best results are obtained, where complicating stricture exists, by curing the latter. Aside from general tonifying, strychnine and ergotine in suppository I have found useful, but I rely most on reducing sensualism, (Bromides) and the application of electricity upon the colliculus seminalis: the Faradic current, as a milder means ; the galvanic, which, of course, cauterizes, as a more formidable weapon. So the treatment is the same as for genuine spermatorrhea. E. H. Fenwick says liquid extract of salix nigra, ℥i, three times a day, checks involuntary emissions, if prostatitis be due to masturbation or excess in venery.

The prostate unmistakably is an organ of the genital apparatus, and empties itself during the orgasm. Its juice excites the spermatozoic activity and lengthens the life of the sperma. This was proven by Fuerbringer, but I find that already long before it was proven, only I forgot where I saw it.

V. Tuberculosis is always a part of the general tuberculosis of the genital tract, and astonishes sometimes through the insignificant effects on the part of the prostate. Treatment, according to general rules.

VI. Atrophy requires no treatment, and is much more frequent in old age than used to be supposed.

VII. Tumors are, except the fibromyomatous, almost always cancerous. Treatment will probably always remain inefficient, although palliation, especially of the hemorrhages, is possible, unless a new efficient cancer remedy should be discovered. Yet, attempts at radical cure have been made. Kuester and Bardenheuer have extirpated sub-peritoneally the prostate and bladder, and implanted the ureters into the rectum, but both patients died after 5 resp. 14 days.

VIII. Stones in the prostate are sometimes formed and felt per rectum, or by the bougie, and have been removed from the perineum or from above, even through the urethra, in a few suitable cases.

IX. Neurosis of the prostate was construed as a separate affection by Home, 1818, and credited to fissures in analogy to fissures of the rectum. I cannot obtain the original work, and so I am left in doubt as to the causation. If such could be ascertained, analogous treatment, as elsewhere in fissures, should obtain, but it seems to me that Home may have seen a neuralgia of the nervous pudendus, as I did, where the flabby penis used to jerk regularly, and where electricity seemed to do some, but little, good.

X. Hypertrophy and fibromyomata. The hypertrophy of the prostate, which generally appears above fifty, but has been seen in babies already, is either due to an increase of the connective tissue, or particularly to the formation of fibromyomata microscopically in perfect keeping with fibromyoma of the uterus, when extreme sizes are met with. The enlargement is in the first case more even, in the latter more lobular; an enlarged lateral or central lobe, or even three enlargements. Such fibromyomata can be shelled, respectively, twisted out of their seat after incision of the mucous membrane. The prostate weighs, normally, ten to fifteen grammes, is about thirty-two mm. long and broad, eighteen mm. thick, and develops suddenly during puberty, but reaches its full size only at twenty-five. It contains about forty glands and ducts. In regard to the value of operative partial removal, the opinions are divided up till today. Already Civiale and Mercier have removed pieces, but as late as 1885 Erichsen writes still that operations were both dangerous and inefficient, and have been almost unanimously condemned. In fact, the sole treatment of enlarged prostate consists in the regular use of the catheter. When prostatic enlargement does not admit catheterization, puncture above the pubes should be made and the canula left in *situ.* Canalization of the prostate is barbarous and correctly abandoned. Brandes (and I may add Leasure, 1855,) punctured through the symphysis pubis, but there is not sufficient evidence of the merits of this procedure. So far I have cited Erichsen.

On the other hand, already in 1852, Demarquay recommended for prostatoliths to incise across the perineum, separate the rectum from the urethra and prostate, incise the latter, and remove the stones, and considers the danger small. I do not speak of the use of medicines as they all proved inefficient. Only Heine claimed results from injections of iodine with Pravaz's needle into the prostate, in 1875, but he found no followers. I tried his plan once without success; the patient died. There Brandt maintains to have seen improvement from massage of the prostate. Thompson and Guyon, two weighty men in genito-urinary diseases, oppose operative measures. The first cites a man who used the catheter for twenty-two years, till he died at ninety, and another who used the catheter on himself 35,000 times. Guyon opposes on the ground that the prostatic enlargement is only a part of a general genito-urinary sclerosis, forming a superabundance of connective tissue in the bladder, and preventing the latter, through loss of muscle, from emptying itself completely, even when the mechanical obstacle is removed, and cites, as sample cases, where the same trouble existed with atrophy of the gland. I am inclined to believe, though, that he dealt with cases of paresis of the bladder from nervous causes. Long rows of other men believe in the efficiency of operative proceedings. Newman, in New York, in momentary cauterizations; Bottini, in burning away the obstructing part. Bruce Clark used Bottini's method, and dropped it again for the suprapubic operation, on account of better seeing. Bottini's results are remarkable. At the Tenth International Medical Congress, August 7, 1890, he reported fifty-seven cases, with two deaths. In thirty-two cases a perfect cure was effected; in eleven, improvement; in twelve, the result was *nil.*

In 1882, Biedert reported five cases of galvanopuncture per rectum, with decided reductions. He used the cathode. Leopold Casper, paper before the Berlin Medical Society, April 18, 1888, used fifteen to twenty-five milliampères for ten to fifteen minutes, and obtained in four cases considerable shrinking of the prostate, with quite an amelioration; thinks, though, that if the hypertrophy is particularly in the middle lobe, that he would not expect much. Of course, he disinfects the rectum before introducing the needle, and the latter is isolated up to the tip. For a test, he made a dog's testicle shrink from walnut down to almond size.

McGill, in Leeds, had from thirty-three suprapubic operations sixteen per cent. deaths.

Mansell Moullin had twenty per cent. from suprapubic; only eight per cent. from perineal operations.

Norton reports four complete successes from the use of his prostatome, introduced by perineal incision.

Tobin used the ecraseur per urethram, guiding the wire with two fingers through suprapubic incision.

B. Schmidt and Moullin saw one recurrence of an operated hypertrophy after nine months. Küster saw, after a partial suprapubic excision, an aggravation of the ability to void the urine spontaneously. On the other hand, he had three decided improvements by operating between rectum and prostate, and excising portions of the lateral lobes. Lately, Dittel reports by this proceeding a fine success. Helferich had no improvement by suprapubic excision in one case. Israel and Bergmann had no lasting improvements suprapubically; Landerer, a splendid one. Esmarch had two excellent successes by chipping out from the perineum, but great losses by pneumonia. The latest sanguine statements for combined suprapubic and perineal operations introduced by M. Schmidt, come from Belfield, who maintains, for about four weeks, a stretched condition of the prostatic urethra, after the combined incision, in the manner of Harrison, by a catheter inserted through the perineal wound. He collected four cases (*American Journal of the Medical Sciences*, November, 1890, page 439,) of combined perineal and suprapubic operations, with one death. Twenty-five per cent. of other forty-one cases of radical operation for which he gathered statistics, voluntary micturition was reëstablished thirty-two times, but only in a few, and he says, it is expressly stated, that the evacuation of the bladder was *complete* as well as voluntary. This is the main hitch. Thompson states that after two years of catheterizing, the bladder will never empty itself *completely*. This is not absolutely true, but very often. The statistics are sadly lacking on this point, as well as many original reports, as not sufficient stress has been heretofore laid upon it, while it is an all-absorbing question, indeed. The question of residual urine is the question of cystitis—remaining permanently or not. No bladder which is not emptied perfectly can be kept free from cystitis, and, if such persists, difficulties of micturition remain, and the cure is imperfect, though voluntary urination is reëstablished. I have seen several prostatics who had to use the catheter, though they were able to urinate, just because of the irritation from residual urine, and the treatment of the cystitis remained without avail, which was promptly successful where residual urine was absent. Even Keyes, who pays attention to this point, neglects to consider it in a number of his published suprapubic and perineal operations. In three cases he obtained perfect emptying of the bladder after suprapubic operations.

If I draw the facit of my reading, I can but join with Keyes's opinions (*Medical Record*, October 31, 1891), the *best* mortality statistics, showing at least 13 6-10 per cent. (Keyes), it is trying to kill a mosquito on a man's nose with a club when you advise early operation, as Belfield does, although I admit that early operations should furnish better statistics. If early, we should operate so early as not to deal with any cystitis or other secondary changes, in order to obtain the advantages of asepsis, which is wellnigh impossible later on. On the other hand, we should not refuse operating in the seemingly most hopeless cases, as some astonishingly good results in such cases are recorded. To gain time, in some cases, we may satisfy ourselves for a while with suprapubic puncture, substituting the trocar for a well-fitting catheter, over which we withdraw the canula. The perineal drainage is more inconvenient. At all events, at the present state of statistics, we are in duty bound to lay the truth, and nothing but the plain truth, before a prostatic we meet, and let him choose for himself. Our patients live only once, and we have no right to take their lives in our hands by their blind permit. They should be enabled to choose intelligently whether to prefer their discomfitures and their final outlook, or the risks of operations with their, after all, so frequently deficient results. The choice of the way to operate will be ofttimes quite baffling. Statistics of suprapubic operations are worse as to the number of deaths, but better in regard to results, if death did not follow. Combined suprapubic and perineal operations admit of the most perfect handling, but the danger to life is, of course, greatest. Want of personal experience makes me shy of expressing an opinion, but I should judge, *a priori*, that Dittel's operation, of separating the rectum and slicing out the requisite amount of prostatic tissue, if it were possible to avoid wounding the urethra, ought to be the ideal, because the most surgical proceeding. Were it not for the profuse bleeding and poor seeing, I would feel certain of my opinion.

The latest for enlargement of the prostate is castration, in analogy of oöphorectomy, for uterine fibroid. Two results are cited by Rocum (*Centralblatt für Chirurgie*, No. 35, 1893). The paper being inaccessible to me at present, I cannot judge the real value from details. White had already shown, in dogs, the shrinkage of the prostate after castration. Civiale saw it accidentally in a lithotomy ; Bilharz and Pelican in eunuchs.

DISCUSSION.

Dr. Tremaine : I labor under some disadvantages in discussing this subject, because I was not aware what points Dr. Hartwig was going to take up. I had no synopsis of his paper ; and to go over the whole ground of diseases of the prostate would take up too much time and weary you. I supposed, however, that the subject he was going to discuss was that which must interest all of us, viz., senile hypertrophy of the prostate. When we come to consider that about one man in fifteen, past middle life, has some enlargement of the prostate, and one man out of perhaps eight or ten, if not a still larger proportion, after fifty-five, has hypertrophy of the prostate in some form, and his life then begins to be somewhat of a burden to him, it is a matter which interests us all most seriously. I fancy that every gentleman in this room has, at some period of his life, and perhaps almost every day of his life, come in contact with just such cases, and has been worried very much to know what to do with them. I confess I share that difficulty with all of you. There is no subject in the whole realm of surgery that is more difficult, and, perhaps, none which now engages the attention of surgeons more generally. Dr. Hartwig has gone over the ground very extensively upon the literature of the subject, and he has collected the views of some gentlemen.

Now, these are conflicting. Guyon, for instance, assumes that this is a part of the degenerative changes that take place in old age, accompanying atheroma, and so on. I do not think that view is tenable, and I will not weary you with going into the reasons why. And I do not agree with Harrison, who is also a distinguished author on this subject, that enlargement of the prostate comes as a result of the pouching of the bladder, and is a sort of hypertrophy of muscle, as it were, from the efforts of the bladder to expel, and goes on increasing in this way, with the residual urine pouching down the bladder behind the prostate, I think a more correct view ; and I speak of this matter of the histology of the subject because a rational understanding of that may lead us into the correct ideas with regard to treatment. I believe the better opinion nowadays, with regard to this, is the opinion which is entertained by many, that enlargement of the prostate gland is analogous with the fibroid of the uterus.

For a long time the prostate gland has been spoken of and thought of as a urinary organ. I am quite satisfied in my own mind that it is not; that it is a genital organ; and, as stated, excessive venery, perhaps, conduces to enlargement of the prostate, although there is one thing that might perhaps militate against that view: that enlargement of the prostate is generally found, or more frequently, in old bachelors than in married men. You can draw your own conclusions.

However, the most important matter for us to consider is, what is to be done. First, the symptoms. We will talk of them a moment. The symptoms may be divided into subjective and objective. One point that I want to make just here is that enlargement of the prostate occurs much earlier than usually taught by the text-books. I have seen it in men between forty and fifty years of age. As a rule, it is said in the books, and by various writers, to occur generally after fifty-five, but I do think that the enlargement commences much earlier than is generally believed. Now, with regard to symptoms. If a patient says he has to get up to urinate frequently during the night, and has no stricture, the presumption is at once in favor of enlarged prostate. If, in addition to that, the urine at times smells badly, I should suggest an examination, and that by the rectum, and the probabilities are that enlarged prostate will be found. But this is somewhat deceptive sometimes, because the enlargement takes place inwards, as it were, against the bladder, and very little enlargement is found in the rectum. I have seen just such a case as that. Now, we go on with our examination, and use an instrument, and that instrument enables us to determine somewhat the nature of the enlargement. If an ordinary catheter passes in without difficulty, and yet there is a difficulty in the outflow of the urine, the chances are that that enlargement is of the valvular character, and I wish I had been able to bring a specimen which once was in my possession, now is no longer, to show this evening, in which there was, at the middle lobe of the prostate, a regular tit-like valve. If that case had been recognized during life and operated on, it would, doubtless, have resulted successfully, because that little valve could have been removed readily by the forceps or by the ecraseur, and the patient relieved. That is one way in which we can determine the nature of the enlargement. If, on the other hand, there is no obstruction to the flow of urine, and you pass an elbow catheter, and that goes in after a time, stops at about seven inches, and then goes in with a jump, the presumption is in favor of enlargement of the middle lobe. If, on the other hand, but a narrow-tail—rat-tail—catheter can be passed in with difficulty, and deflects to one side or the other, the presumption is in favor of enlargement of the lateral lobes. Now, the first two forms of enlargement are those most amenable to treatment, in my judgment. It has been my lot to see a great many of these cases, and I am seeing them almost every day.

With regard to treatment, when there is difficulty in urinating and frequency of urinating during the night, and some amount of residual urine in a man otherwise healthy, I think careful catheterization, to remove the residual urine, is the best mode of treatment; but that involves the most scrupulous care with regard to cleanliness of the catheter, and is almost impossible of attainment. It is almost impossible to impress upon the patient himself that scrupulous cleanliness which is so necessary to prevent cystitis. Now, so long as you can keep clear of cystitis, and so long as you can remove this residual urine by catheterization, I should certainly advise against an operation; but there will almost certainly come a time when catheterization becomes difficult, when its results are not so good as they were previously; in other words, when the relief afforded is not so great, and then the question of operation must come up, because that is the golden moment. If you allow that to pass, you will soon have cystitis, septicemia, pyemia, uremia, if you choose, and death. I can cite you many instances that have occurred in this city, under my observation, where I have urged operation. One, the case of a medical man that was well known to us all here, and who resisted the operation, allowed the opportunity to pass, and the results followed which always do in such cases—uremia and death.

Now, with regard to the choice of operation—the kind of operation. Some ten years ago, I had the honor to invite the attention of the medical profession in America to suprapubic cystotomy. At that time, the operation was barely alluded to in our various textbooks. It had not then been done even by some of the great genito-urinary surgeons of the world, notably such as Sir Henry Thompson, and by none of any eminence or distinction in America. It was reserved for cases of very large stone, as a last resort. At that time, in the American Surgical Association, great objection was urged against it. One gentleman, Dr. Roberts, of Philadelphia, made the prediction, and stated that he desired to place himself on record—that before ten years that, viz., suprapubic cystotomy, would be the received operation. His prediction has been more than verified. The suprapubic operation is now being done wholesale by almost all surgeons of this country, and done very successfully by Dr. Hunter McGuire, of Richmond, in just such cases as this, enlarged prostate; and he reports the most remarkable successes. Dr. Belfield, of Chicago, whose work I am familiar with and have observed a good deal of it, has been remarkably successful too. The suprapubic route, I think, will readily commend itself to you all, because you have the advantage of inspection, and you can reach any portion of the bladder, or any portion of the prostate, with your finger. The perineal route has some advantages, but a surgeon must have a much longer forefinger than I have if he can reach the prostate; and I do not think one man in a thousand has a forefinger with which he can reach an enlarged prostate by the perineal route and be able to make that dilatation which is necessary. By the perineal route, of course, catheterization has to be kept up for some length of time, and the dread of hemorrhage is not so very great, because a tube can be introduced and surrounded by what the French call the *chemise a canule*, which is made of iodoform gauze, and hemorrhage arrested in that way without difficulty. But the suprapubic operation enables you to inspect every portion of the bladder, to reach every portion of the bladder, and, if prostatectomy is necessary, to perform it with very little risk. Suppose, for instance, the enlargement be of the middle lobe, the enlarged part of the prostate projecting can be readily pinched off with the forceps, the vesical orifice dilated with the forefinger, and the patient can, in all probability, get immediate relief. But suppose that the lateral lobes are enlarged, and lapped over in this way, just as my thumbs here, lapping over that way (indicating), why, of course, there it becomes a more difficult operation, and to excise a portion of the prostate there, is a somewhat serious undertaking, on account of the hemorrhage. That objection has been met, though, very beautifully by Dr. Keyes, of New York, by packing with iodoform gauze the bladder and carrying a catheter through the urethra, attaching a thread, and drawing that thread out through the urethra, so that the bladder can be packed twenty-four hours, and hemorrhage arrested that way by antiseptic gauze, or iodoform gauze preferably. So that objection is removed. Now, this has been successfully done.

Dr. Hartwig referred to Moullin's tables. I think his later tables give a percentage of 14.3, about the same as by the perineal route. That shows that increased skill is brought about by increased experience, as in many other forms of surgical operation. I will not weary you with reciting cases, but there was one very notable case that I operated on some four or five years ago, where the patient suffered intensely from enlarged prostate—a man eighty-two years of age. I did the suprapubic section, and established an opening there, and had made at that time a little apparatus, which I will show you, which has since been improved on by an Eastern surgeon, Dr. Gerish, I think, of Portland, Me. This is a little hard rubber drainage-tube, with a flange and cap, —which was made for me by an electrician in this city—held in place by an elastic band, and was worn successfully by the old gentleman for several years afterward. He was a patient of Dr. Callahan's. I am not sure whether he is alive now, but he was, three or four years after, in comparative comfort. I think Dr. Phelps assisted me in the operation. You remember the case, Doctor?

Dr. Phelps: Yes.

Dr. Tremaine: Now, I have had five other cases—none of that advanced age. A remarkable point of that case is the advanced age, and the complete comfort that was given the old gentleman. Dr. Hunter McGuire establishes a fistulous opening there, and does away with the necessity of the tube; but I think this is an advantage, using an instrument of this kind. I think I may claim to be the originator of this instrument, although it has since been modified and improved by Dr. Gerish, of Portland. I wish Dr. Hartwig had quoted, if it is familiar to him, a paper written recently by Dr. White, of Philadelphia, who has traversed this whole subject thoroughly, and has elaborated one view, that which was based on the analogy of these tumors of the prostate, or enlarged prostate, with hypertrophy of the uterus, and suggested by reduction of hypertrophy of the uterus after removal of the ovaries.

White made a series of very elaborate experiments on dogs, and demonstrated beyond question that in dogs the prostate shrunk after castration; and it has been suggested since that castration was not necessary, but that ligature of the spermatic vessels would accomplish the same purpose. I regret that I did not know this when I was in Egypt last Winter, or I should have made some inquiries with regard to the effect that was found upon eunuchs, but I have already written to Dr. Keating, vice-president of the medical college there, and asked him if he would gather some statistics on that subject, which I hope in due time to receive. Hunter observed this fact long ago with regard to the shrinkage of the prostate gland in animals, and the enlargement of it during the rutting season, so that there seems to be good ground for Dr. White's view in regard to that. Now, as to the advisability of doing this. When one considers the suffering, if it is once demonstrated that that will produce the effect, there can be no question of the advisability of doing it, because there is scarcely any man that would not be perfectly willing to part with the testicles to get relief from the intense suffering these cases involve. There might be a great deal said on the subject of palliative treatment, a good deal of caution given with regard to the use of the catheter, and the dangers of cystitis as the result of it; but, as the hour is already late and I have taken up a good deal of your time, I will make way for others.

Dr. William H. Heath: With respect to Dr. Hartwig's paper, like Dr. Tremaine, I did not know what was to be talked about. Dr. Hartwig has covered the whole ground of prostatic diseases. Some statements are not in accord with my experience. In the first place, as to prostatic abscesses. I have seen quite a large number; I have never seen one open through the perineum. I have seen them open internally and into the rectum, and have opened them into the rectum myself, but I have never known them to open elsewhere. The removal of the prostatic gland is anatomically possible; surgically, it is unjustifiable. Referring to the most important and the common affection of the prostatic gland, hypertrophy, there is one part of the treatment that has not been considered—namely, the preventive and early treatment of prostatic obstruction in cases of enlargement of the gland. Harrison, of London, who has just been quoted, has made prominent the preventive treatment of obstruction by mechanical dilatation. It consists in the "maintenance of the waterway," as he calls it, by the systematic passage of soft bougies, ironing out, or molding away, as it were, the encroaching mass. The time to institute this is in earliest stage, when frequency of micturition, and particularly at night, indicates that the tumor is beginning to interfere with urination. Its object is to lessen venous congestion, and, therefore, irritation, and keep the urinary level, thus averting the serious chain of consequences so likely to ensue. A slight obstruction does not preclude its employment. The value of this procedure has demonstrated itself to me many times, and should be tried in every suitable case.

Castration, as a remedy for enlargement of the prostate, is, as yet, an unknown factor. It would seem, however, that when the conditions were such that a man would be willing to part with these organs, it would be too late to be of benefit, while, on the other hand, few would be willing to make the sacrifice for dangers yet remote and amenable to other methods. It would be valuable to know how soon and to what extent atrophy follows. Subcutaneous division of the vas deferens has been tried.

The question of operation for prostatic obstruction arises in numerous cases where catheterization is no longer possible. By what route and what should be done are each in themselves enough for discussions. The suprapubic, with perineal drainage, is the accepted one. In general, too much should not be attempted in the old and feeble, as many of these cases are. Drainage alone often gives satisfactory relief. The medical treatment of these cases should not be overlooked by any means. By regulating the diet habits, and keeping the urine mild and aseptic by internal administration of salol, boracic acid, etc., cleanliness, gentleness in use of catheter, comfort may be had and the dreaded days postponed. Among the better class, many become more expert than any surgeon in manipulating their own cases.

While the importance of gentleness and patience in instrumentation in prostatic as well as stricture cases is generally understood, cases yet frequently come into the hospital lamentably punished. It seems almost impossible to some to resist the inclination to use force.

Dr. W. C. Phelps: I have very little to say on this subject; Dr. Hartwig has so thoroughly covered the ground. I have about come to the conclusion that Dr. Tremaine has spoken to you about. I have had occasion, several times, to make suprapubic cystotomy in cases that have come into the hospital, or that I have taken there, and up to this time I am sure that I have not used any other method, and have not really known any other method to offer any sort of chance of relief. That it is a very great relief, I am absolutely certain. I have had patients that were utterly miserable in every sort of way, where they had reached a point where catheterization was a very painful procedure, where pus was constantly flowing along the catheter, sometimes where it would flow out from an over-distention of the bladder, with septic conditions, etc. Take a case like that and make suprapubic cystotomy. The patient lies quietly in bed, and, once in a while, is able to get up and go around with an apparatus similar to that which Dr. Tremaine has shown. I have had patients live some time, but I have never yet run across a case where my patient was able to walk around and draw off his urine, as Dr. Tremaine mentions in that old Irishman. I saw him, and it was certainly a very great relief to him, and a very remarkable and successful case. Those I have had have not been so mild as that; all of them have had pus. I hardly think that there was any pus in that patient of Dr. Tremaine's; I am not quite certain; but where pus has formed in the bladder I most always observe more or less kidney trouble, or something of that sort, and the patient is pretty apt to pretty soon die.

I had a case, last Summer, of an abscess of the prostate, which came out in a curious way. It was a case of appendicitis, that came into the hospital, on which I operated for a very immense abscess, reaching down into the pelvis. The patient got along very well at first, and then the temperature commenced to run up again, with a difficulty of passing the bowels. I put my finger into the rectum, to see if there was anything there, and I found an immense swelling of the prostate. That was excised, and I drew, probably, a pint of pus.

Then, there is one case, I remember, which was a very remarkable case of enlarged prostate. I never saw one so marked or so large. There was a rim all the way around the internal opening of the urethra, that is, the neck of the bladder. I think it stood up, at least, an inch from the wall of the bladder, entirely around. It was not an enlargement of the middle lobe. It seemed to be an enlargement of the whole gland, and the only way any operation could have relieved that man would have been by tunneling down through the lower part of it. It would have been absolutely impossible for the urine to have got over that rim; it seemed to project at least an inch into the bladder.

Dr. Mynter: In regard to the treatment of those cases of old gentlemen with the difficulty in passing urine, its getting offensive in smell, and, at last, these other complications—the question is what to do under those circumstances. Last Summer I got a most serious hemorrhage in an old gentleman, seventy-six years of age, with an enlarged prostate, and who had catheterized himself for years. The bleeding occurred and recurred, and was almost clear blood. I syringed it out, and used iodine and gave ergot, and injected alum and acetate of lead. Then, I got the idea one day—why couldn't we use antipyrin, as, perhaps, the hemorrhage was temporary in character and capillary ; and I applied a twenty per cent. solution of antipyrin, and it checked the hemorrhage then and there. I used that in three other cases, and in each case the hemorrhage was stopped short by the use of antipyrin in a twenty per cent. solution. If this is useful in capillary bleeding, and stops, for instance, bleeding of the nose and bleeding from the brain in operations on the brain, I see no reason why it should not be applicable in such cases as this.

In regard to the operation itself, there is none easier in the whole range of surgery than suprapubic cystotomy ; that is, on condition that the bladder will contain six or eight ounces of water, and that you have access through the rectum. First, I generally dilate the bladder ; it makes it a great deal easier. And I want to call your attention to the fact, that it is still easier if you inject air into the bladder instead of water. That is a question that has been brought up lately, I have forgotten by whom, in cases of suprapubic cystotomy—simply fill the bladder with air. The air is more elastic, and does not produce rupture so easily. As far as the extirpation of the prostate itself is concerned, I have never done that operation. I have made dozens of cystotomies probably, but they were for other reasons—cancer of the bladder, for instance, stone, and so on, and in one for a peculiar case of stricture.

Dr. Rochester : There are one or two points I would like a little information on. Dr. Tremaine, in his remarks, said that where there was frequent urination in a man over forty years of age, it was a very probable indication of enlarged prostate. It seems to me that the first thing I would think of under such conditions would be an investigation of the reaction of the urine, as to whether that frequent urination might not be due to acid.

I have in mind three cases of men between forty and fifty who presented this symptom of frequent urination, particularly at night, which has been corrected by simply correcting the acidity of the urine.

The next point that came to my mind was this matter of the operation of castration for the cure of enlarged prostate, and the fact that dogs had been operated upon and it had been shown that the prostate had markedly decreased in size. I want to ask whether it is known whether the dogs that were operated on were old dogs or young dogs, and whether that would not have some bearing upon the matter of the prostate. In men in whom the prostate is enlarged to a great extent the function of the testicle is largely gone, and the question arises whether in those circumstances the removal of the testicle would have any effect whatever in reducing the enlargement of the prostate. Of course, I can understand that if the prostate be enlarged in younger men, where the function of the testicles is markedly present, that it might have some effect upon the reduction of that swelling; but in old men, in whom the function of the testicles is largely gone, would it have any effect in reducing the size of the prostate?

Dr. THORNBURY: Along the line of Dr. Rochester's remarks, I desire to accentuate the factor of sexual excess as a causative factor in enlargement of the prostate; and, in doing that, I simply expound the views of an eminent Southern surgeon, who has had opportunities for a very extended observation among colored men, in whom we all know sexual excess is very frequent; and he claims that sexual excess is, par excellence, the cause of enlargement of the prostate, and that the enlargement takes place before the function of the testicle is materially impaired, and is the direct cause of the trouble; and Dr. White's experiments go to fully substantiate this fact, of which he gave a very elaborate account here last summer, at the meeting of the American Surgical Association.

Dr. HEATH: Dr. Tremaine said that enlarged prostate was not quite so common with young men as with old men, and that is very true. That is quoted by a good many authorities. There is no question about it, that every man that has ever lived has committed sexual excesses. There is no question about the relation between sexual excesses and enlarged prostate. There is no relation. That is an exploded idea. In fact, the prostate gland is not a gland at all, but a muscle, and the hypertrophy might just as well come from emissions and from sexual excesses. The fact of sexual excesses I do not think has anything to do with it. I do not think we know why it occurs any more than we know why tumors occur in colored women in the uterus; not a bit. We do not know the action at all.

Dr. Hartwig: Of course I have to refute Dr. Tremaine's statement that I did not mention White's experiments on dogs. That was the very last thing I said. I am sorry that I cannot answer Dr. Rochester, because I did not see the original account by White, as to whether those dogs were tested with regard to their age. Indeed, this is a question of pertinence, I think, as regards our conclusion concerning men, because it is just as doubtful to my mind as it is whether castration in old age will have the same effects as castration at an earlier age.

The effects of antipyrin, as alluded to by Dr. Mynter, are certainly very gratifying in all cases of bleeding, and I should suppose that it would have a satisfactory effect in the bladder too. It is certainly in keeping with what I know of the effects of the antipyrin.

But the essence of what I should have liked to hear from the gentlemen, I did not hear, and that is what the opinion is in regard to operating on cases which show only the premonitory symptoms —the summons to urinate too often at night—simple indications which cause us to examine for enlargement of the prostate, and where we do find enlargement, while cystitis does not as yet exist, and while we know that this very man will, within five, or six, or eight years, have serious trouble. There it is really difficult to give advice; there it is a little difficult to form an opinion; and there it is where I could not form an opinion, in spite of studying the whole literature of the subject. This is a thing which I think the future must elaborate. The statistics are at present, of course, too meager to admit of forming an opinion. So I can but say that I come to the conclusion, after hearing what all the gentlemen have said, that the conclusions of my paper, the original conclusions, are correct.

Dr. Tremaine: I would like to have an opportunity. I think it is very important to answer Dr. Hartwig upon one question, if the Academy will permit. I do not wish to take up the time, but I think the matter is too serious to be passed over.

Dr. Hartwig has asked a question which I thought I answered. I thought I said that when catheterization could be done, and there was no impairment of the general health, and the patient could go right along, and once or twice, or three or four times a day, remove the residual urine, when there was still no cystitis, when the health was unimpaired, and practical comfort could result from that, that was no time for operative interference; but there came a time when that relief did not come, and when there was difficulty in getting relief in that way, and that before cystitis came on in a marked degree, or subsequent renal changes or general impairment of health, then was the time for operation ; and there can be no question, I think, about that. So that if Dr. Hartwig is in doubt, I think that clearly answers the point, and I think that this is the general consensus of the best surgeons who have studied this class of cases. I am called almost every day of my life to see such cases ; and I advise that operation. The difficulty is to get patients to consent to it. Only within the last week or two I urged upon an old gentleman that he have this operation done. He put it off, and put it off, under the advice of his attending physician, the general practitioner — who will go along and temporize, and temporize, and temporize, until the golden opportunity is gone, and then send their patients in awful suffering into their graves, when a little operation, timely done, would give them years of comfort and life, as I have proved and demonstrated again and again.

Now, I think the question can be answered, and it is answered ; and if anybody is in doubt about it, let them call me in in their practice.

